# Transcriptomic Profiles of Senegalese Sole Infected With Nervous Necrosis Virus Reassortants Presenting Different Degree of Virulence

**DOI:** 10.3389/fimmu.2018.01626

**Published:** 2018-07-17

**Authors:** Alejandro M. Labella, Esther Garcia-Rosado, Isabel Bandín, Carlos P. Dopazo, Dolores Castro, M. Carmen Alonso, Juan J. Borrego

**Affiliations:** ^1^Departamento de Microbiología, Facultad de Ciencias, Universidad de Malaga, Malaga, Spain; ^2^Departamento de Microbiología y Parasitología, Instituto de Acuicultura, Universidad de Santiago de Compostela, Santiago de Compostela, Spain

**Keywords:** *Solea senegalensis*, reassortant nervous necrosis virus, transcriptome, RNA-Seq, differentially expressed genes

## Abstract

Betanodaviruses [nervous necrosis virus (NNV)] are the causative agent of the viral encephalopathy and retinopathy, a disease that affects cultured Senegalese sole (*Solea senegalensis*). NNV reassortants, combining genomic segments from redspotted grouper nervous necrosis virus (RGNNV) and striped jack nervous necrosis virus (SJNNV) genotypes, have been previously isolated from several fish species. The wild-type reassortant wSs160.03, isolated from Senegalese sole, has been proven to be more virulent to sole than the parental genotypes (RGNNV and SJNNV), causing 100% mortality. Mutations at amino acids 247 (serine to alanine) and 270 (serine to asparagine) in the wSs160.03 capsid protein have allowed us to obtain a mutant reassortant (rSs160.03_247+270_), which provokes a 40% mortality decrease. In this study, the RNA-Seq technology has been used to comparatively analyze Senegalese sole transcriptomes in two organs (head kidney and eye/brain) after infection with wild-type and mutant strains. A total of 633 genes were differentially expressed (DEGs) in animals infected with the wild-type isolate (with higher virulence), whereas 393 genes were differentially expressed in animals infected with the mutant strain (37.9% decrease in the number of DEGs). To study the biological functions of detected DEGs involved in NNV infection, a gene ontology (GO) enrichment analysis was performed. Different GO profiles were obtained in the following subclasses: (i) biological process; (ii) cellular component; and (iii) molecular function, for each viral strain tested. Immune response and proteolysis have been the predominant biological process after the infection with the wild-type isolate, whereas the infection with the mutant strain induces proteolysis in head kidney and inhibition of vasculogenesis in nervous tissue. Regarding the immune response, genes coding for proteins acting as mediators of type I IFN expression (*DHX58, IRF3, IRF7*) and IFN-stimulated genes (*ISG15, Mx, PKR, Gig1, ISG12, IFI44, IFIT-1*, to name a few) were upregulated in animals infected with the wild-type isolate, whereas no-differential expression of these genes was observed in samples inoculated with the mutant strain. The different transcriptomic profiles obtained could help to better understand the NNV pathogenesis in Senegalese sole, setting up the importance as virulence determinants of amino acids at positions 247 and 270 within the RNA2 segment.

## Introduction

Senegalese sole (*Solea senegalensis*, Kaup 1858), a flatfish belonging to the *Soleidae* family, presents a high importance in the European aquaculture due to its fast growth rate in captivity and to its high commercial value (1). Nowadays, the main limiting factor for Senegalese sole culture is the appearance of microbial epizootic outbreaks, being the viral encephalopathy and retinopathy (VER) or viral nervous necrosis one of the most important, since disease outbreaks achieve almost 100% of mortality in cultures of sole (2).

Nervous necrosis virus (NNV) is the etiological agent of VER, affecting more than 40 marine and freshwater fish species worldwide (2, 3). NNV (genus *Betanodavirus*, family *Nodaviridae*) is a non-enveloped icosahedral virus with two single-stranded positive-sense RNA segments (RNA1, 3.1 Kb, encodes the RNA-dependent RNA polymerase; RNA2, 1.4 Kb, encodes the capsid protein) (4, 5). A subgenomic segment [RNA3, 0.4 kb, encoding the non-structural proteins B1 and B2, has been described by several authors (6–8)]. Based on the variable T4 region (RNA2), NNV has been classified into four species: striped jack nervous necrosis virus (SJNNV), tiger puffer nervous necrosis virus, redspotted grouper nervous necrosis virus (RGNNV), and barfin flounder nervous necrosis virus (BFNNV) (3). Moreover, several RGNNV-SJNNV reassortant isolates, presenting both genomic segment combinations, SJNNV/RGNNV and RGNNV/SJNNV, have been isolated from several fish species (9–11). A highly virulent RGNNV/SJNNV reassortant, harboring a RGNNV-type RNA1 and SJNNV-type RNA2 segments, has been isolated from Senegalese sole, and named wild-type (wt) isolate (wSs160.03) (10). Compared to SJNNV genotype, this viral reassortant isolate displays sequence differences affecting amino acidic positions 247 and 270 at the C-terminal extreme of the capsid protein, which seems to be related to the increase of its virulence (12, 13). In fact, a mutant of this viral reassortant obtained by reverse genetic, presenting substitutions of serine to alanine at amino acid position 247 and of serine to asparagine at residue 270 (named rSs160.03_247+270_), causes a reduction in sole mortality of 40% compared to the wt isolate (14).

RNA-Seq technology is a sequencing-based method that allows to study the entire transcriptome in a high-throughput and quantitative manner, becoming the primary technology to be used for gene expression profiling (15). One of its major advantages is that it can capture transcriptome dynamics across different tissues or conditions without sophisticated normalization of data sets (16). Therefore, this technology provides valuable data for understanding virus–host interactions, and it has been used to study pathogenic processes during fish virus infection (17–21). This information is essential to understand fish immunity against microbial pathogens, and to develop effective strategies to prevent fish diseases.

The aim of the present study is to identify differentially expressed genes (DEGs) involved in Senegalese sole response against infections with NNV reassortants with different degrees of virulence. RNA-Seq technology has been used to obtain the transcriptomic profiles from Senegalese sole head kidney (one of the major lymphohaematopoietic organs in fish) and nervous tissues (pools of eye and brain, the NNV target organs) after infection with the wt isolate wSs160.03, and the mutant strain rSs160.03_247+270_. Different types of vaccines have been tested in various fish species relevant to the aquaculture sector (22). However, no vaccine for Senegalese sole against NNV has been reported. The results obtained provided relevant information about sole–NNV interaction and, therefore, for the development of new strategies to control this viral disease.

## Materials and Methods

### Virus and Cell Culture

The betanodaviruses used in this study were the wt isolate (wSs160.03) and the mutant strain harboring pinpoint mutations at position 247 and 270 of the capsid protein (rSs160.03_247+270_) (10, 14). Both viral strains present a combination of segments RGNNV (RNA1)/SJNNV (RNA2). The GenBank accession numbers for wSs160.03 (SpSs-IAusc160.03) genome segments are FJ803911 (RNA1) and FJ803923 (RNA2). Viruses were propagated on E-11 cells (23) in Leibovitz medium (L-15, Gibco, Paisley, UK) supplemented with 2% fetal bovine serum (FBS, Gibco) and 0.5% penicillin/streptomycin (Sigma-Aldrich, St. Louis, MO, USA). Inoculated cells were incubated at 25°C for up to 15 days. Cell culture supernatants were recovered after the appearance of cytopathic effects, and clarified by centrifugation at 5,000 × *g* for 10 min at 4°C. Viral suspensions were stored at −80°C. Viral titrations were performed in 96-well plates (Nunc, Thermo Electron LED GmbH, Wiesbaden, Germany) using the 50% tissue culture infectious dose (TCID_50_) method (24).

### Experimental Infections and Sampling Procedures

Three groups of 30 Senegalese sole juvenile specimens (5–10 g) were acclimatized for one week at the aquarium services of the University of Malaga (Spain). Animals were fed with a commercial diet (Biomar, Palencia, Spain), and maintained in 100-L aquaria with aeration under stable temperature (18 ± 0.5°C) and salinity (33–35 g/L). Fish used in this study have been treated according to the Guidelines of the European Union Council (Directive 2010/63/EU) and the Spanish directive (RD 53/2013). Three experimental groups were considered: (A) negative control, injected with L-15 Leibovitz medium; (B) animals injected with the wt isolate, wSs160.03; and (C) animals injected with the mutant strain, rSs160.03_247+270_. Animals from the three groups were intramuscularly injected (IM), and the viral dose used in the groups B and C was 2 × 10^5^ TCID_50_/fish. Three fish per group were randomly collected at 24, 48, and 72 h post-inoculation (p.i.), and were euthanized by a MS-222 (Sigma-Aldrich) overdose. Individual samples of head kidney and pooled eye/brain were aseptically recovered, and immediately frozen in liquid nitrogen, and stored at −80°C until used.

To minimize fish suffering, trials were accomplished in accordance to the Spanish directive (RD 1201/2005) for the protection of animals used in scientific experiments, and by the Bioethics and Animal Welfare Committee of the IFAPA for the regulation of animal care and experimentation (approved number 10-06-2016-102).

### RNA Extraction and cDNA Synthesis

Organs collected (50–100 mg) from inoculated fish were homogenized in 1 mL of TRI Reagent^®^ (Sigma-Aldrich) using a mixer mill MM400 (Retsch, Haan, Germany) with a program of 20 f/s 5 min. After 5 min at room temperature, a volume of 100 µL of 1-bromo-3-chloropropane (Applichem, Darmstadt, Germany) was added to the samples and vortexed for 15 s. After 5 min at room temperature, samples were centrifuged at 12,000 × *g* for 15 min at 4°C, obtaining three phases. The upper aqueous phase was recovered and mixed with ethanol 75% (1:1). To continue the extraction of total RNA, RNeasy Mini kit (Qiagen GmbH, Hilden, Germany) was used according to the manufacturer’s instructions. RNA samples were resuspended in nuclease-free water (Qiagen), quantified by spectrophotometry at 260 nm using a NanoDrop ND 1000 Spectrophotometer (NanoDrop Technologies, West Palm Beach, FL, USA), and stored at −80°C until used. RNA samples were treated with RNase-free DNase I (Roche Diagnostics GmbH, Mannhein, Germany), to avoid genomic DNA contamination, following manufacturer’s instructions. For the synthesis of cDNA, Transcriptor First Strand cDNA Synthesis Kit (Roche) was used following manufacturer’s instructions. cDNA samples were quantified at 260 nm with the nanodrop system, and stored at −20°C until used.

### Sample Preparation for RNA-Seq and Raw Reads Data Processing

RNA-Seq was performed using the Illumina HiSeq™ 2000 platform (Centro de Análisis Genómico, CNAG-CRG, Barcelona, Spain). To select the time-point for RNA-Seq analysis, transcription of *Mx* gene and viral replication were quantified using previously described amplification protocols (25, 26). RNA quality/quantity was checked following the criteria proposed by CNAG-CRG, including a RNA integrity number RIN > 8 (Agilent 2100 Bioanalyzer, Agilent, Waldbronn, Germany), and absorbance ratios 260/280 and 260/230 of 1.8–2.0 (Figure S1 and Table S1 in Supplementary Material). RNA-Seq was performed to obtain > 40 million paired-ended (PE) reads of 75 bp length per sample. RNA-Seq raw reads (FASTQ) (27) were processed using bioinformatics tools of the Supercomputing and Bioinnovation (SCBI) center of the University of Malaga. Data were temporally stored at the Picasso SGI Origin 2000 supercomputer (SCBI).

Pre-processing of raw reads was performed using the SeqTrimNext (v2.0.60) pipeline (SCBI) in order to remove low quality sequences, contaminants, adaptors, vectors, and other artifacts prior to the assembly (28). The script bowtie2 (v2.2.9) (29) was used for mapping and assembling the pre-processed FASTQ reads using as reference the Senegalese sole transcriptome database SoleaDB, v.4.1 2014-01-23^1^ (30). Finally, the script sam2counts (v20131126) (31) was used to convert SAM mapping results into reference sequence counts.

These RNA-Seq data have been deposited in the NCBI Gene Expression Omnibus database with the experimental series accession number: GSE101877.

### Statistical Analysis, Identification, and Annotation of DEGs

To identify DEGs, cleaned, mapped, and counted data sets obtained from experimental groups B and C (animals inoculated with wSs160.03 and rSs160.03_247+270_ reassortants, respectively) were compared to the control group A (animals injected with L-15 medium). The Bioconductor package (including edgeR, DESeq2, and limma approaches), which uses R statistical programming language,^2^ was applied for the analysis and comprehension of high-throughput transcriptomic data (32–35). The false discovery rate (FDR) was used to determine the threshold *p*-value for multiple tests (34). Transcripts with FDR values lower than 0.05 (significance level) were considered as DEGs. Venn-Diagram method was used to compare and visualize datasets of experimental groups with the three different Bioconductor approaches (36). For the functional annotation of the transcripts, Sma3s, AutoFact, and Full-LengtherNext were used to provide gene description (37–39). These annotation tools were consulted from SoleaDB transcriptome database (30). In addition, Gene Ontology (GO) enrichment analysis was carried out using GO database^3^ and genes with fold change (FC) values higher than 1.5 (either up- or downregulated).

### Analysis of Gene Expression by Quantitative Real-Time PCR (qRT-PCR)

All the reactions were conducted using the LightCycler 96 Termocycler (Roche) and the Fast Start Essential DNA Green MasterMix (Roche) using SYBR Green technology. PCRs were carried out in 20-µL mixtures containing cDNA generated from 50 ng of RNA, 10 µL of Fast Start Essential DNA Green Master 2×, 1 µL of each primer (10 pmol) and 7 µL of water. Termocycling conditions were: initial denaturation at 95°C for 10 min, followed by 45 amplification cycles of 95°C for 10 s, 60°C for 10 s, and 72°C for 10 s. To obtain melting curves, the following profile was conducted: 95°C for 10 s, 65°C for 60 s, and 97°C for 1 s. Each sample was run in triplicate for analysis. Eight differentially expressed genes were randomly selected for qRT-PCR verification. Ribosomal protein subunit 4 gene (*rps4*) was stably expressed throughout the experiments and was used as the internal control (Table S2 in Supplementary Material). The relative expression ratio of the target genes versus *rps4* gene was calculated using 2^−ΔΔCT^ method, and all data were given in terms of relative mRNA expression (40).

## Results

### Transcriptomic Sequencing and Raw Reads Data Processing

Transcription of *Mx* gene and viral replication were quantified in both head kidney and eye/brain samples from each experimental condition (L-15, wSs160.03, and rSs160.03_247+270_) at 24, 48, and 72 h p.i. Transcription of *Mx* and viral replication reached the highests values at 48 h p.i., and neither *Mx* transcription nor viral replication was observed at 24 h p.i. (data non-shown). Therefore, samples from 48 h p.i. were selected for RNA-Seq analysis (threefold replicated), being sequenced using Illumina HiSeq™ 2000 platform. Between 269,346,896 and 331,555,320 raw reads were obtained from head kidney samples. These reads were pre-processed with the SeqTrimNext pipeline, and between 2.3 and 3.1% of contaminant sequences were removed (Table 1). In eye/brain samples, the range of raw reads obtained was between 289,923,568 and 310,151,374. After the SeqTrimNext pre-processing, 20% of contaminant sequences were removed (Table 1). After the mapping and assembling of the pre-processed reads with bowtie2 script, using as reference the SoleaDB transcriptome (v.4.1 2014-01-23), a total number of 56,089 unigenes (28,954 from head kidney and 27,135 from eye/brain samples) were obtained for the group infected with the wt, high virulent isolate (wSs160.03). Regarding the group infected with the mutant, less virulent strain (rSs160.03_247+270_), 56,455 total unigenes (28,964 from head kidney and 27,491 from eye/brain samples) were obtained (Table 1). These results represent a difference on transcript detection of 0.65% between animals inoculated with both viruses.

**Table 1:** Illumina sequencing analysis of tissue Senegalese sole samples infected with nervous necrosis virus reassortants, and L-15-control group, at 48 h p.i.

Sample	Total raw reads^a^	Total clean reads^b^	% contaminant sequences removed	Total unigenes^c^
**Head kidney**				
L-15	331,555,320	321,395,622	3.1	–
wSs160.03	269,346,896	263,149,296	2.3	28,954
rSs160.03_247+270_	303,553,076	296,703,761	2.3	28,964
**Eye/brain**				
L-15	289,923,568	231,003,910	20.3	–
wSs160.03	310,151,374	245,611,206	20.8	27,135
rSs160.03_247+270_	293,997,192	238,197,453	19	27,491

*^a^Total number of read-pairs (or reads for single-end runs) that passed Illumina filter*.

*^b^Total number of clean reads after the analysis with the SeqTrimNext pipeline*.

*^c^Total number of unigenes after mapping with bowtie2 using Solea DB (v.4.1 2014-01-23) as reference transcriptome*.

### Identification of Differentially Expressed Genes

To identify DEGs, transcripts detected from animals inoculated with the wt or the mutant strains were compared with those obtained from the control group (L-15), using three statistical approaches of the Bioconductor package (edgeR, DESeq2, and limma). The analysis with edgeR was the most efficient method, detecting 1,026 DEGs (99.5% of 1,031 total DEGs) (alone or together with DESeq2) (Table 2), whereas with the application of DESeq2, only 359 DEGs (34.8%) (alone or together with edgeR) were obtained (data not shown). No DEGs were obtained with the limma tool.

**Table 2:** DEGs recorded with edgeR from *Solea senegalensis* subjected to viral infection with nervous necrosis virus reassortants at 48 h postinfection.

	Upregulated genes (%)	Downregulated genes (%)	Total DEGs	FC^a^ ratios (up-/downregulated)
**wSs160.03**				
Head kidney	358 (88)	49 (12)	407	14.2–12.3
Eye/brain	206 (91)	20 (9)	226	5.2–3.2
Total	564 (89)	69 (11)	633	
**rSs160.03_247+270_**				
Head kidney	129 (93)	10 (7)	139	11.6–12.4
Eye/brain	28 (11)	226 (89)	254	5.3–3.5
Total	157 (40)	236 (60)	393	

*^a^Range of fold-change values for each reassortant and organ sampled*.

Based on the results obtained with edgeR, a total of 633 DEGs were detected in animals inoculated with the wt reassortant (highly virulent), whereas 393 DEGs were detected in animals infected with the mutant strain, which means a reduction of 37.9%. Regarding up- and downregulated DEGs, the percentages between up- and downregulated genes were 89 and 11%, respectively for the wt isolate, while for the mutant strain, the percentages were 40 and 60%, respectively (Table 2). According to the organs tested, the percentages of up- and downregulated DEGs in head kidney was similar for both viral strains, obtaining 88 and 12% for wSs160.03, and 93 and 7% for rSs160.03_247+270_ (Table 2). For the eye/brain samples, a similar proportion between up-/downregulated DEGs was obtained after infection with the wt isolate (91 and 9%); however, an inversion in the up-/downregulated DEGs proportion (11 and 89%) was obtained in eye/brain samples from animals inoculated with the mutant strain (Table 2).

### Comparative Analysis of DEGs Detected After Infection With Both Viral Reassortants

**Figure 1:**
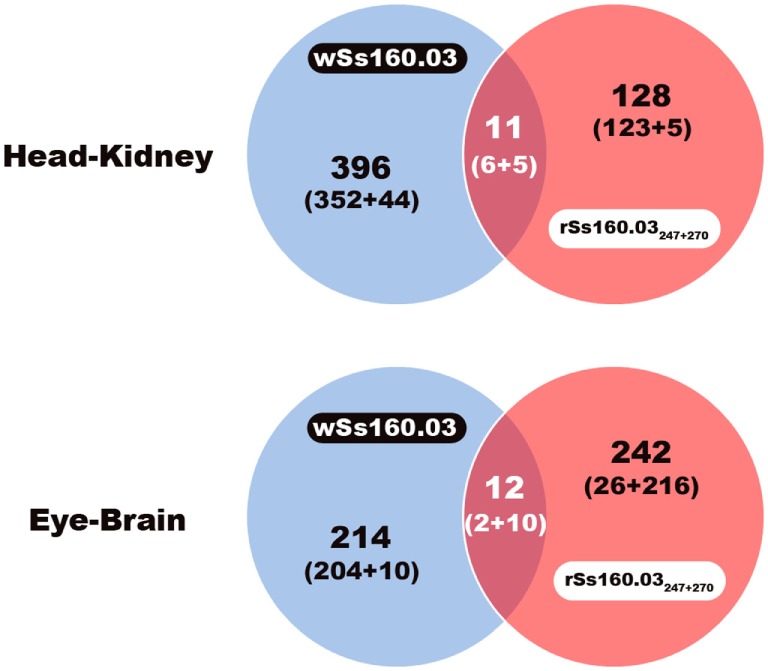
Venn diagram of DEGs using edgeR. Common and different DEGs detected after infection with the wild-type and mutant reassortants in head kidney and nervous tissue. Number of DEGs: upregulated + downregulated.

A comparative analysis has been performed in order to determine shared and different DEGs detected between the infection with the wt and the mutant strains. From the total number of DEGs detected after infection with both viral strains (1,026), 610 DEGs (60.8%) were exclusively detected after the infection with the wt isolate, whereas 370 DEGs (36.9%) were exclusively detected after infection with the mutant strain. The number of shared DEGs obtained between both viral strains was only 23 (2.3%) (Figure 1), mainly related to functions such as, signal transduction pathways, inflammatory response, cellular integrity, and metabolism (Table S3 in Supplementary Material).

Comparing DEGs detected from head kidney samples, 396 and 128 DEGs were exclusively identified after the infection with the wt and mutant strains, respectively; whereas, the number of shared DEGs detected was only 11, being 6 upregulated and 5 downregulated (Table S3 in Supplementary Material). In the case of eye/brain samples, a similar number of DEGs were identified after the infection with wt and mutant strains (214 and 242, respectively). The number of shared unigenes deregulated was only 12, being 2 upregulated and 10 downregulated (Figure 1).

### Functional Annotation

**Figure 2:**
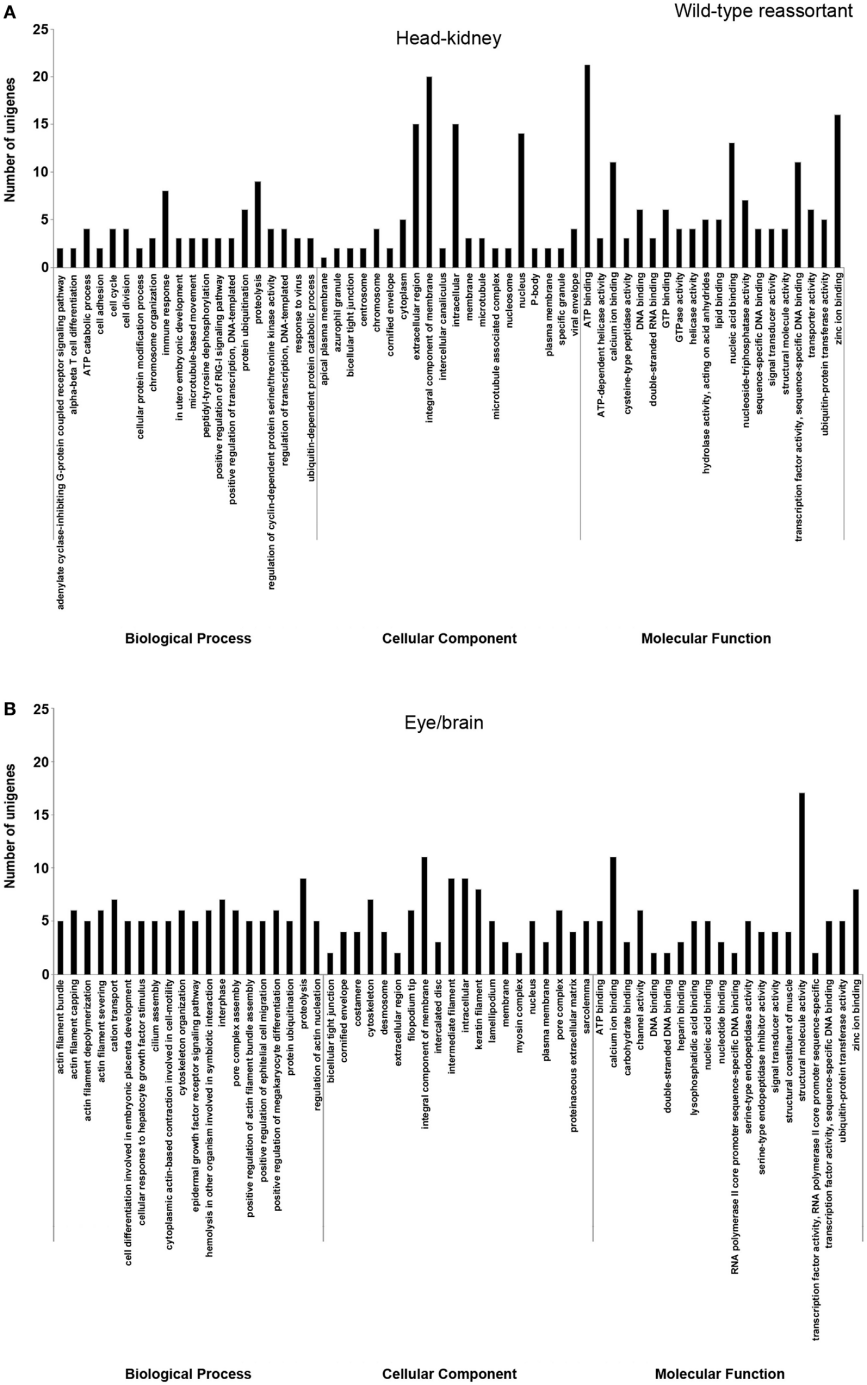
Gene ontology enrichment analysis of differentially expressed genes from head kidney **(A)** and eye/brain **(B)** samples of Senegalese sole animals infected with the wild-type reassortant. Ontologies were classified into three subclasses, including biological process, cellular component, and molecular function.

**Figure 3:**
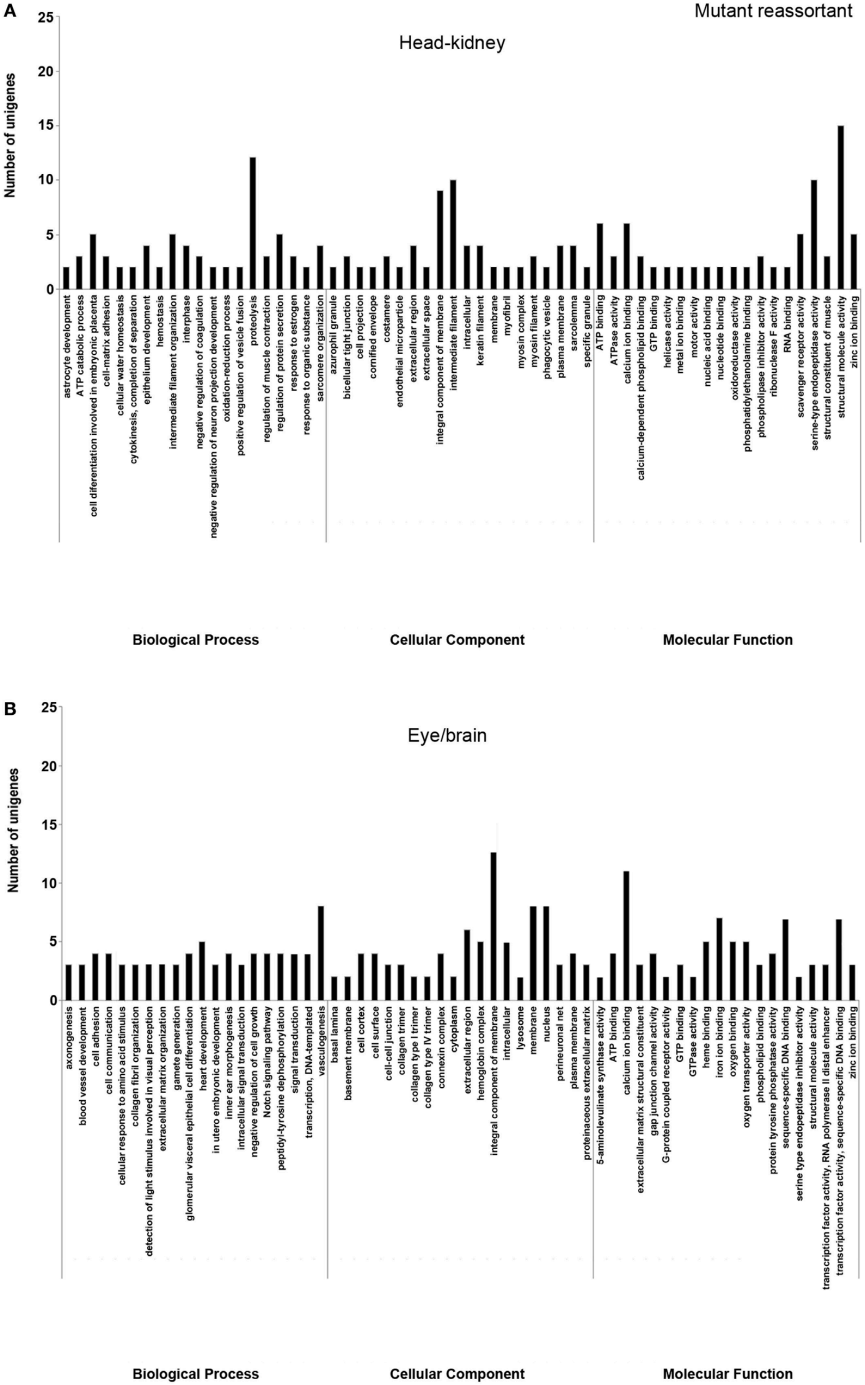
Gene ontology enrichment analysis of differentially expressed genes from head kidney **(A)** and eye/brain **(B)** samples of Senegalese sole animals infected with the mutant reassortant. Ontologies were classified into three subclasses, including biological process, cellular component, and molecular function.

In order to study the biological functions of the identified DEGs, a GO enrichment analysis was performed. Only unigenes with FC higher than 1.5 (up- or downregulated) and *p*-value < 0.05 were considered. For the wt reassortant infection samples, a total of 530 unigenes were detected (319 in head kidney and 211 in eye/brain samples). In contrast, in samples infected with the mutant strain, lower numbers of unigenes (296) were obtained (135 and 161 in head kidney and in eye/brain samples, respectively) (Table 3). Different percentages of GO database annotation for the unigenes were obtained for the samples analyzed, with a reduction of 10.25% of annotated unigenes in the mutant strain infection samples (Table 3). However, the number of associated ontologies for each unigene was higher in the mutant infected samples. DEGs were classified into three subclasses: biological process (BP), cellular component (CC), and molecular function (MF), and the 20 ontologies more frequently detected within each subclass are represented in Figures 2 and 3, for the wt and mutant strains, respectively.

**Table 3:** Annotated unigenes in gene ontology database.

	Total number of unigenes^a^	Number of annotated unigenes (%)	Number of different ontologies detected
**wSs160.03**			
Head kidney	319	269 (84.33)	411
Eye/brain	211	113 (53.55)	282
Total	530	382 (72.07)	693
**rSs160.03_247+270_**			
Head kidney	135	73 (54.07)	202
Eye/brain	161	110 (68.32)	566
Total	296	183 (61.82)	768

*^a^Total number of unigenes with fold change higher than 1.5 (up- or downregulated)*.

Different ontology profiles were obtained for each type of viral infection. This result was specially evidenced for the BP subclass; specifically, in head kidney samples, only “proteolysis” and “ATP catabolic process” were shared in both viral infection samples, while no similarities were observed in samples from eye/brain. In the case of CC and MF subclasses, similar profiles were detected between both viral infection samples. The more frequent ontologies within CC and MF subclasses in both organs sampled, had a clear relation to cellular membrane and extracellular matrix with an energetic cost associated (“ATP binding”), and to calcium and zinc-dependent enzymatic activities (Figures 2 and 3).

Regarding each viral infection, in head kidney samples inoculated with the wt reassortant isolate, the most frequently detected ontologies were “proteolysis” (BP), “integral component of membrane” (CC), and “ATP binding” (MF). In eye/brain samples, the profile obtained was more homogeneous, varying only “structural molecule activity” in the MF subclass (Figure 2). For the samples inoculated with the mutant strain, the most predominant ontologies were “proteolysis” (BP), “intermediate filament” (CC), and “structural molecule activity” (MF) in head kidney samples; and “vasculogenesis” (BP), “integral component of membrane” (CC), and “calcium ion binding” (MF) in eye/brain samples (Figure 3).

The unigenes associated with the most frequently detected ontologies within the BP subclass, “proteolysis,” and “vasculogenesis” are detailed in Table S4 in Supplementary Material. Regarding “proteolysis,” 18 unigenes (9 in each organ) were deregulated in animals infected with the wt isolate (FC values from −1.67 to 9.04 in head kidney and from 1.62 to 4.30 in eye/brain samples), whereas 15 unigenes (12 in head kidney and 3 in eye/brain samples) were deregulated in the case of the mutant strain (FC values from 2.46 to 9.11 in head kidney and from −2.40 to 2.49 in eye/brain samples) (Table S4 in Supplementary Material). Cysteine proteases, serine proteases, and metalloproteases were the main enzyme families detected. In contrast, “vasculogenesis” was only detected in eye/brain samples of animals infected with the mutant strain. For this ontology, eight unigenes were detected, being all downregulated with FC values ranging from −2.69 to −1.53 (Table S4 in Supplementary Material).

### Immune Response of Senegalese Sole Against Viral Reassortant Infections

Shared DEGs in samples of both organs tested h were identified after infection with both viral strains. For the wt isolate, 42 DEGs (6.6% out of 633) were detected in both organs. Most of these DEGs were upregulated, except for *COL1A2*, which is involved in inflammatory response and signaling pathways and, which was downregulated in both organs. Regarding upregulated DEGs, a clear relationship with the innate immune response against viral infection was observed, being unigenes detected those coding pattern recognition receptors (PRRs) (*DHX58*), and IFN-stimulated genes (*ISG15, Mx, STAT1, HERC5, IFI44, IFIT-1, NUP133*, and *TRIM21*). In addition, unigenes related to the following cellular functions were also upregulated: (i) apoptosis and cell proliferation (*MACPF, u-PAR, PARP14, EPSTI1*, and *ISG12*); (ii) antigen processing and presentation (*RNF213, HERC4*, and MHC class II genes); (iii) signaling pathways (*ANXA3* and *RTP3*); (iv) inflammatory response (*CCL19L1*); and (v) cytoskeleton and extracellular matrix (*SMCHD1* and *ACTB*) (Table S5 in Supplementary Material). Interestingly, only 3 DEGs (0.8% out of 393) (*COL1A2, RDH13*, and *RPS12*), were detected in samples of both organs after inoculation with the mutant strain, all of them downregulated (Table S5 in Supplementary Material) and related mainly to signaling pathways and inflammatory response.

The analyses of genes expressed after infection with each of viral isolate also allow us to detect upregulation of type I IFN (*IFN I*) genes exclusively in animals infected with the wt isolate, including genes encoding for PRRs, mediators of IFN signaling cascades, and IFN inducible proteins (Table S6 in Supplementary Material). In addition, other genes related to the antiviral response have also been upregulated (FC > 10) in head kidney samples of Senegalese soles inoculated with the wt isolate, such as genes related to protein ubiquitination (*MAGEL2*, FC: 11.08); antigen processing and presentation (*GILT*, FC: 11.08); virus responsive genes (VGR) (*Herpes gp2 multi-domain protein*, FC: 11.08; *claudin-like protein ZF-A89*, FC: 10.21); inflammatory response genes (*4F2 cell-surface antigen heavy chain-like*, FC: 9.9); immune effectors (*cathepsin Z*, FC: 9.04); and genes related to apoptosis (*BIRC5*, FC: 11.22; *rho GTPase-activating protein 11A*, FC: 7.75; and *v-ets erythroblastosis virus E26 oncogene homolog 1*, FC: 8.27) (Table S6 in Supplementary Material). Furthermore, several unigenes related to C-type lectins were mainly identified in the nervous tissue (Table S6 in Supplementary Material).

In contrast, the mutant strain did not induce DEGs related to IFN I neither in head kidney nor in nervous tissue; however, DEGs related to C-type lectins recognition and signaling mechanisms were upregulated in head kidney samples. In addition, several unigenes with a role in the immune response of Senegalese soles were detected, especially those involved in different signaling pathways (JNK, TLRs, protein ubiquitination, G-protein), and inflammatory response (Table S7 in Supplementary Material). These DEGs were downregulated in eye/brain samples, unlike it was observed after infection with the wt isolate, where most of the DEGs were upregulated.

### Verification of DEGs by qRT-PCR

**Figure 4:**
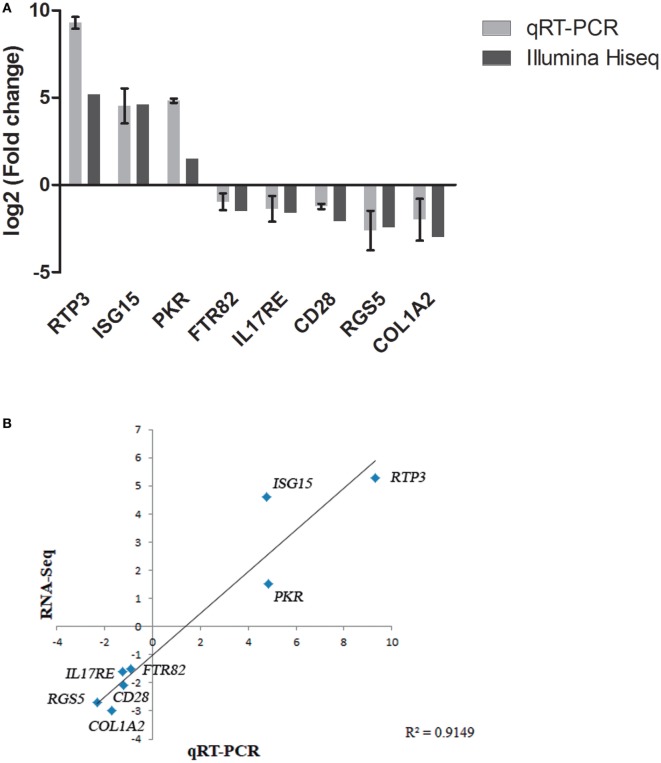
Validation of RNA-Seq data by quantitative real-time PCR (qRT-PCR). **(A)** Comparison of the expressions profile of eight DEGs determined by Illumina HiSeq™ 2000 sequencing platform and qRT-PCR at 48 h (pi) using ribosomal protein subunit S4 (*rps4*) as housekeeping gene. Data shown are the mean of triplicates ± SD. **(B)** Correlation of data between RNA-Seq and qRT-PCR techniques.

To further confirm the transcriptomic sequencing, the expression profiles of eight randomly selected DEGs (including genes related to different pathways, up or downregulated, detected after infection with both viral strain, and both organ sampled) were measured by qRT-PCR. As shown in Figure 4, the qRT-PCR results revealed similar expression tendency as the high-throughput sequencing data, despite some quantitative differences at the expression level, confirming that expression of DEGs detected by RNA-Seq is happening. In addition, unigenes up and downregulated were adjusted with an *R*^2^ = 0.91 demonstrating a good correlation between both techniques.

## Discussion

Immune response against betanodavirus infections has been poorly studied. In order to improve the knowledge of betanodavirus pathogenesis in Senegalese sole, two viral strains with different degree of virulence have been tested, wSs160.03 a natural RGNNV/SJNNV reassortant highly virulent to Senegalese sole (14), and a lower virulent isolate, the mutant reassortant (rSs160.03_247+270_) harboring mutations at positions 247 and 270 in the capsid protein. These mutations resulted in decay of 40% in the fish mortality rate, indicating that these amino acidic positions would have an important effect on virulence (14, 41). In this study, we have applied the RNA-Seq technology to study the possible role of these virulence determinants in the immune response of Senegalese sole juveniles.

This is the first study that applies a RNA-Seq massive sequencing strategy to obtain the whole transcriptome of Senegalese sole after viral infections. In fact, as far as we know, until now, there are just two studies applying RNA-Seq to determine Senegalese sole transcriptome (30, 42). However, none of these authors studied the implication of viral infections on Senegalese sole transcriptome.

The methodology used for pre-processing and assembling the Illumina raw reads allowed us to detect almost the same number of unigenes (differences lower than 1%) for each viral reassortant and tissue analyzed, which provides a high degree of consistency in the subsequent comparative analyses performed. A slightly lower number of unigenes was detected in nervous tissue (eye and brain), probably because of the high percentage of contaminated sequences removed from this tissue (Table 1) that could be due to the own nature of the tissue.

A higher number of DEGs was detected after infection with the high virulent reassortant isolate (Table 1). Purcell et al. (20), using isolates with high- and lower-virulence of infectious haematopoietic necrosis virus, reported that the most virulent isolate induced a higher host transcriptomic change in rainbow trout (20). Furthermore, the proportion of shared genes induced by both viral reassortant strains was quite low (2.3%) (Figure 1), compared with the number of genes expressed differentially by both reassortants. It is also remarkable that after the inoculation with the mutant strain, the number of DEGs was lower in head kidney samples, being the number of downregulated genes higher in nervous tissues (Table 2). The inversion in the proportion of up/downregulated DEGs observed in nervous tissue, the NNV target organ, after infection with the mutant strain could support the hypothesis that mutations at aminoacid 247 and 270 provoke conformational changes in the viral capsid that could result in a loss of affinity for the host cell receptors or in a different tropism of the virus (41).

The ontology analyses indicate variability between the types of viral strains and tissues. Thus, the different number of ontologies detected for both reassortants takes special relevance if we compare them with the number of total unigenes detected for each viral strain. This result could reflect a more specific host response to infection with the wt isolate (693 ontologies associated with 530 unigenes) in contrast to that observed for the mutant strain (768 ontologies associated with 296 unigenes) (Table 3). The main ontologies detected into the subclasses of CC and molecular function for both viral strains were “integral component of membrane,” “ATP binding,” “zinc ion binding,” “Ca ion binding,” to name the most frequently recorded. Although there is an initial energy-dependent recognition of the viral pathogen through the fish cellular membrane by means of Ca^2+^ and Zn^2+^, the signal transduction mechanisms triggered after this initial process are completely different in both reassortants, deriving in the activation of different biological processes in the host cell. The conformational changes provoked by the mutations at amino acids 247 and 270 of the viral capsid may result in the induction of different transductional pathways, leading to different biological process: immune response and proteolysis for the wt isolate, and proteolysis and vasculogenesis inhibition for the mutant reassortant.

It is known that NNV causes apoptosis in its host (43). In our study, cell apoptosis, identified as proteolytic cleavage of cellular proteins, was detected in animals infected with the wt isolate, as well as in head kidneys of animals infected with the mutant strain. The proteolytic cleavage includes the participation of cathepsins, serine proteases, calpains, and metalloproteases. The infection with the mutant strain provokes downregulation of genes related to vasculogenesis in nervous tissue, provoking inhibition of vasculogenesis, and further apoptosis (44, 45). These results indicate that both viral strains induced apoptosis, although through different mechanisms.

Differentially expressed genes and ontology analyses evidence that immune response is a biological process mainly regulated in head kidney of fish inoculated with the wt isolate, highlighting the innate immune response mediated by IFN I. Viral nucleic acid is detected in the host cells by PRRs, being RLR and MyD88-dependent TLR signaling pathways found to mediate type I IFN induction in response to RNA virus infection (46). The RLR pathway is composed of three RNA cytoplasmic sensors, such as RIG-I, MDA5, and LGP2. In the present study, only *LGP2* (also named *DHX58*) gene was upregulated in head kidney and nervous tissues after infection with the wt isolate, whereas no deregulation of *RIG-I* or *MDA5* was observed in any experimental group. No detection of *RIG-I* has been recorded in gilthead seabream (*Sparus aurata*) and European seabass (*Dicentrarchus labrax*) after infection with betanodaviruses (47); in fact, it has been described that the presence of *RIG-I* is limited to ancient fish (48). Upregulation of *LGP2* together with *MDA5* has also been detected in European seabass after infection with betanodaviruses (47). *RIG-I* and *MDA5* have a caspase recruitment domain (CARD), which leads to IFN expression, and confers an antiviral state (49). *LGP2* lacks the CARD domain; however, it seems to be implied in modulating *RIG-I* and *MDA5* signaling (50–52). MyD88 serves as an adaptor protein for downstream signaling of mammalian TLRs. TLR3 recognizes viral double-stranded RNA through TRIF, whereas TLR7 and TLR8 can detect single-strand RNA (ssRNA) virus through MyD88 signaling. In the present study, only *TLR3* was slightly upregulated (FC 1.05) (data not shown) in head kidney samples after the infection with the wt isolate infection, at least at the time point tested.

Upregulation of interferon regulatory factors *IRF3* and *IRF7* has also been detected in head kidney samples, but no deregulation was recorded for *MAVS, TRAF3, TANK*, and *TBK1*, genes involved in IFN I signaling pathway. This result correlates with the absence of any deregulation of *RIG-I* and/or *MDA5*, which are responsible for the activation of this signaling cascade through the CARD domain. Furthermore, it has been described that an innate immune response could be triggered with the activation of *TBK1* without involvement of *IRF3* and *IRF7* (53) and, on the contrary, it has been described that gilthead seabream is able to clear NNV infection without upregulation of *TBK1*, suggesting that *TBK1* is not essential for the innate immune response (54). Interestingly, *IRF3* and *IRF7* were only upregulated in head kidney samples after infection with the high virulent reassortant; however, *ISG15* and *Mx* were upregulated in both tissues analyzed. This could be explained by the existence of different antiviral defense mechanisms; thus, recent studies suggest that mammalians have antiviral defense systems that act before the induction of IFN I (55). In addition, no deregulation of IFN I was observed in any sample at the time analyzed, which could be due to the fact that IFN expression is very fast and punctual (47). Induction of the IFN I pathway after infection with the wt isolate was also evidenced by the upregulation of several interferon-induced proteins (*IFIT-I, ISG15*, and *IFI44*) in head kidney and nervous tissue, and some exclusively in head kidney (*GIG1* and *interferon-induced very large GTPase 1*).

Chemokines are a family of cytokines having a major role in trafficking and activating immune response of leukocytes (56). Little is known about the expression of Senegalese sole chemokines after viral infections. The results obtained in the present study are similar to those reported by Carballo et al. (57), who described the upregulation of several chemokines in this fish species after infection with lymphocystis diseases virus, an iridovirus (57). Regarding other flatfish, a few chemokines and chemokine-receptors have been cloned and characterized from turbot (*Scophthalmus maximus*), tongue sole (*Cynoglossus semilaevis*), and Japanese flounder (*Paralichthys olivaceus*) (58–61), including CCL19, a CC chemokine that induce migration of head kidney leukocytes and increases host immune response in turbot (61). In the present study, CCL19 has also been found upregulated in both organs after the infection with the high virulent virus (Table S6 in Supplementary Material).

A possible innate immune mechanism mediated by C-type lectins has been detected especially in nervous tissue of animals infected with the wt isolate (Table S6 in Supplementary Material). Ourth et al. (62) described a host protective mechanism against channel catfish herpesvirus by binding to host mannose binding-lectin (62), a C-type lectin, and activation of the complement-mediated lectin pathway. On the other hand, it is also known that enveloped viral pathogens can exhibit highly glycosylated proteins being able to attach to C-type lectin receptors that could be used as an entry mechanism into the host cell evading the innate immune response, hijacking the host cell biosynthesis machinery and allowing virus replication and dissemination (63). However, it should be taken into account that betanodaviruses are non-enveloped viruses and, although Chi et al. (64) described grouper nervous necrosis virus (GNNV) as a glycosylated virus (64), there are not further evidence to support this hypothesis. In fact, Chen et al. (65) described three glycerol (GOL) and one polyethylene glycol molecules into the P-domains of the GNNV capsid protein, being the latest responsible for the enhancement of GNNV attachment to heparin sulfate proteoglycans (HSP) (65); however, the presence of glycoproteins have not been described to date. Therefore, the role of C-type lectins against infections by NNV highly virulent to Senegalese sole is unknown, and further studies are needed.

Regarding Senegalese sole animals infected with the mutant strain, no IFN I effectors were deregulated, being regulated different signaling routes and defensive cellular mechanisms (Table S7 in Supplementary Material). Furthermore, a downregulation of all DEGs was observed in nervous tissues, inhibiting processes like inflammation and vasculogenesis in the brain cells.

In conclusion, the highest virulent viral isolate provokes a higher transcriptomic change, involved mainly genes related to immune response and proteolysis, most of them upregulated. On the contrary, mutations at positions 247 and 270 provoke conformational changes in the viral capsid, which are translated into different transductional mechanisms, leading to downregulation of genes in nervous tissues, mainly related to inflammation and vasculogenesis. The host immune response against the mutant strain is moderate and no induction of the IFN I antiviral system has been detected.

## Ethics Statement

Fish used in this study have been treated according to the Guidelines of the European Union Council (Directive 2010/63/EU) and the Spanish directive (RD 53/2013). To minimize fish suffering, trials were accomplished in accordance to the Spanish directive (RD 1201/2005) for the protection of animals used in scientific experiments, and by the Bioethics and Animal Welfare Committee of the IFAPA for the regulation of animal care and experimentation (approved number 10-06-2016-102).

## Author Contributions

EG-R and JB conceived and designed the study. AL carried out all gene expression experiments and data analyses. AL, DC, and MA performed the experimental trials. AL and EG-R wrote the manuscript. JB revised the manuscript. IB and CD supplied the viral reassortant strains and revised the manuscript.

## Conflict of Interest Statement

The authors declare that the research was conducted in the absence of any commercial or financial relationships that could be construed as a potential conflict of interest.

## Footnotes

^1^www.scbi.uma.es/soleadb (Accessed: April, 2017).

^2^https://www.bioconductor.org/ (Accessed: April, 2017).

^3^www.geneontology.org (Accessed: April, 2017).

## Supplementary Material

Figure S1 Agarose gel electrophoresis of total RNA extracted for RNA-Seq technology. **(A)** Head kidney samples. From left to right: control group (L15-R1, -R2, and -R3), wild-type reassortant (RG160/SJ160-R1, -R2, and -R3), and mutant reassortant (RG160/SJ160 m247 + 270-R1, -R2, and -R3). **(B)** Eye–brain samples. From left to right: control group (L15-OC1, -OC2, and -OC3), wild type reassortant (RG160/SJ160-OC1, -OC2, and -OC3), and mutant reassortant (RG160/SJ160 m247 + 270-OC1, -OC2, and -OC3). Molecular weight marker VII (Roche-Diagnostics).
