# Supplementary figures and images for: Transcriptomic Profiles of Senegalese Sole Infected With Nervous Necrosis Virus Reassortants Presenting Different Degree of Virulence
Source: Front Immunol. 2018 Jul 17;9:1626. doi: 10.3389/fimmu.2018.01626 (PMC6056728; doi:10.3389/fimmu.2018.01626)

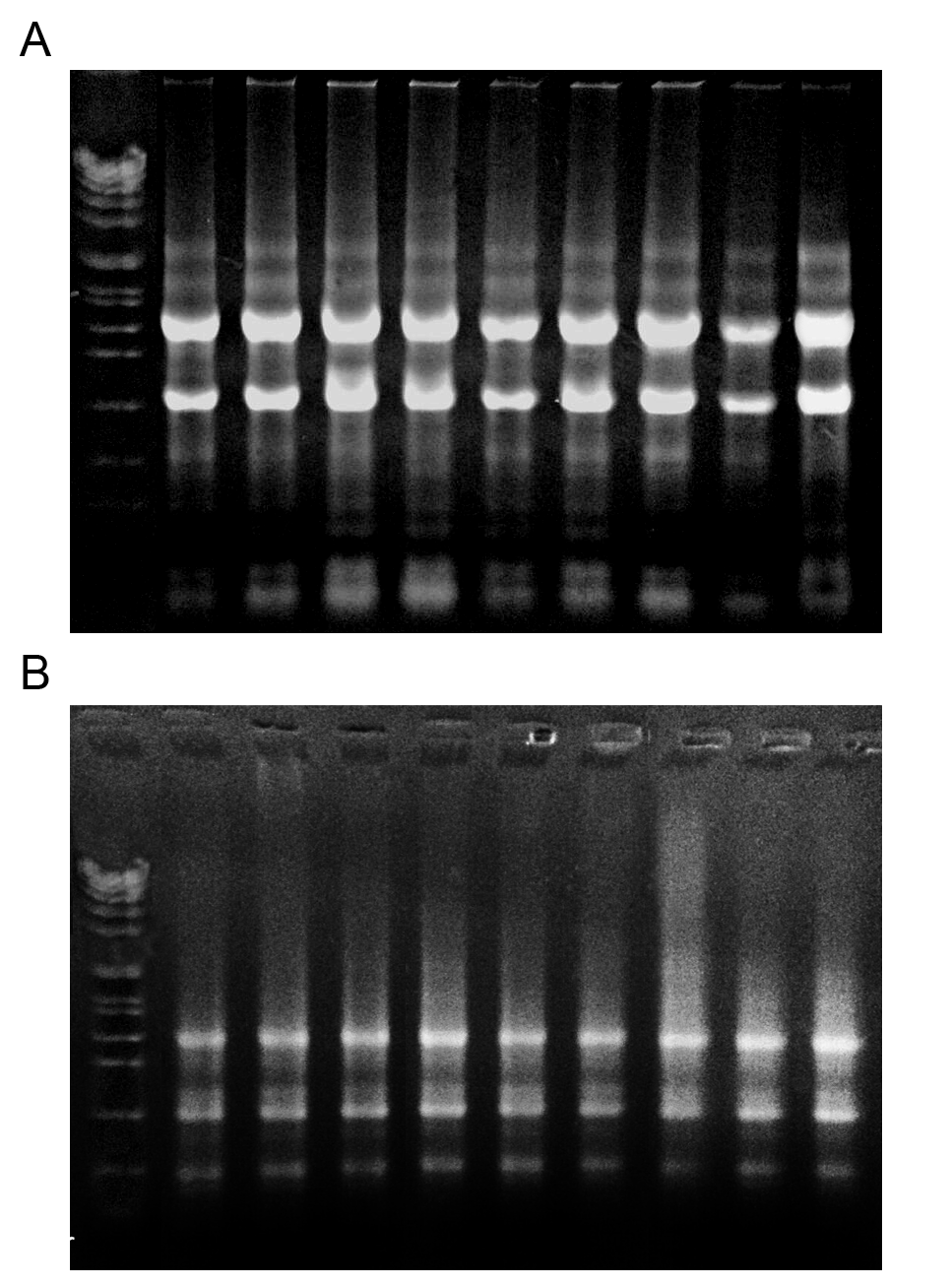

Supplement: Figure S1 — Agarose gel electrophoresis of total RNA extracted for RNA-Seq technology. (A) Head kidney samples. From left to right: control group (L15-R1, -R2, and -R3), wild-type reassortant (RG160/SJ160-R1, -R2, and -R3), and mutant reassortant (RG160/SJ160 m247 + 270-R1, -R2, and -R3). (B) Eye–brain samples. From left to right: control group (L15-OC1, -OC2, and -OC3), wild type reassortant (RG160/SJ160-OC1, -OC2, and -OC3), and mutant reassortant (RG160/SJ160 m247 + 270-OC1, -OC2, and -OC3). Molecular weight marker VII (Roche-Diagnostics). [file Image_1.tif]
