# Supplementary material for: Transcriptomic Profiles of Senegalese Sole Infected With Nervous Necrosis Virus Reassortants Presenting Different Degree of Virulence
Source: Front Immunol. 2018 Jul 17;9:1626. doi: 10.3389/fimmu.2018.01626 (PMC6056728; doi:10.3389/fimmu.2018.01626)
Supplement: Supplementary file 2 [file Table_1.docx]

Supplementary Table S1. Total RNA quality/quantity profiles of Senegalese sole samples used for RNA-Seq.

| Samples | CNAG^a^ barcode | Material source | ng/µl | Absorbance ratio 260/280 | Absorbance ratio 260/230 | BA RIN^b^ | BA ribosomal ratio |
| --- | --- | --- | --- | --- | --- | --- | --- |
| L15-R1 48H | AB8507 | Kidney | 766.7 | 2.07 | 2.48 | 10.0 | 1.6 |
| L15-R2 48H | AB8508 | Kidney | 671.7 | 2.05 | 2.40 | 10.0 | 1.6 |
| L15-R3-48H | AB8509 | Kidney | 1669.2 | 2.09 | 2.41 | 10.0 | 1.6 |
| RG160/SJ160-R1-48H | AB8510 | Kidney | 1574.4 | 2.08 | 2.38 | 10.0 | 1.6 |
| RG160/SJ160-R2-48H | AB8511 | Kidney | 954.3 | 2.08 | 2.44 | 9.9 | 1.5 |
| RG160/SJ160-R3-48H | AB8512 | Kidney | 1134 | 2.07 | 2.31 | 10.0 | 1.6 |
| RG160/SJ160 m247+270-R1-48H | AB8513 | Kidney | 1586 | 2.08 | 2.39 | 10.0 | 1.8 |
| RG160/SJ160 m247+270-R2-48H | AB8514 | Kidney | 845.1 | 2.07 | 2.31 | 10.0 | 1.6 |
| RG160/SJ160 m247+270-R3-48H | AB8515 | Kidney | 1786.3 | 2.07 | 2.35 | 10.0 | 1.6 |
| L15-OC1 48H | AC0885 | eye/brain | 184.4 | 2.01 | 2.11 | 7.9 | 1.6 |
| L15-OC2 48H | AC0886 | eye/brain | 241.3 | 2.03 | 2.17 | 8.1 | 1.8 |
| L15-OC3-48H | AC0887 | eye/brain | 199.5 | 2 | 2.15 | 8.3 | 1.9 |
| RG160/SJ160-OC1-48H | AC0888 | eye/brain | 278.6 | 2.02 | 2.21 | 8.6 | 1.9 |
| RG160/SJ160-OC2-48H | AC0889 | eye/brain | 204.3 | 2.01 | 2.07 | 7.9 | 1.6 |
| RG160/SJ160-OC3-48H | AC0890 | eye/brain | 221.7 | 2.03 | 2.23 | 8.1 | 1.7 |
| RG160/SJ160 m247+270-OC1-48H | AC0891 | eye/brain | 224.8 | 2 | 1.95 | 8.2 | 1.7 |
| RG160/SJ160 m247+270-OC2-48H | AC0892 | eye/brain | 187.3 | 2.02 | 2.23 | 8.2 | 1.7 |
| RG160/SJ160 m247+270-OC3-48H | AC0893 | eye/brain | 208.8 | 2.02 | 2.24 | 8.4 | 1.7 |

^a^CNAG: Centro Nacional de Análisis Genómico

^b^RIN: RNA Integrity Number
