# Supplementary material for: Transcriptomic Profiles of Senegalese Sole Infected With Nervous Necrosis Virus Reassortants Presenting Different Degree of Virulence
Source: Front Immunol. 2018 Jul 17;9:1626. doi: 10.3389/fimmu.2018.01626 (PMC6056728; doi:10.3389/fimmu.2018.01626)
Supplement: Supplementary file 3 [file Table_2.docx]

| Gene name | Forward primer (5’-3’) | Reverse primer (5’-3’) | Amplicon size (bp) |
| --- | --- | --- | --- |
| *RTP3* | CACCCAGTGTCGAAGAACCT | TGATTCTGGGATCCACCATT | 149 |
| *ISG15* | GGGCCTGAACATACTCCAAA | ACCATTGGTGCTTGGTCTTC | 121 |
| *PKR* | TAACACGTCGACCCAGTCAA | GACAACCTTGACGGCGTAAT | 130 |
| *FTR82* | TACATTTGTCCCCAGTGCAA | GGACGTGTTGAGGTTGAGGT | 123 |
| *IL17RE* | TGAAAGCCTTGTGACAGCAC | GGGCTGCAGAGAAACAAGTC | 118 |
| *CD28* | TGTGTGGAAGCAGGACTCAC | CGACATCCAGCAAAGAGTCA | 144 |
| *RGS5* | AAAATGCCATCGACCTCATC | CTGGCCATAGCTGTTGTTGA | 137 |
| *COL1A2* | TCAGGGAATTCCAGGAGATG | TCCAGGTTCTCCATCAGGTC | 106 |
| *RPS4* | CACCCAGTGTCGAAGAACCT | TGATTCTGGGATCCACCATT | 103 |
| Receptor-transporting protein 3 (*RTP3*), Interferon-stimulated gene 15 (*ISG15*), Double-stranded RNA activated protein kinase (*PKR*), FinTRIM family, member 82 (*FTR82*), Interleukin-17 receptor E-like (*IL17RE*), T-cell-specific surface glycoprotein CD28 (*CD28*), Regulator of G-protein signaling 5 (*RGS5*), Collagen type I alpha-2 (*COL1A2*), Ribosomal protein subunit 4 (*RPS4*). | | | |

**Supplementary Table S2.** Primers used in this study for validation of RNA-Seq data.
