# Supplementary material for: Transcriptomic Profiles of Senegalese Sole Infected With Nervous Necrosis Virus Reassortants Presenting Different Degree of Virulence
Source: Front Immunol. 2018 Jul 17;9:1626. doi: 10.3389/fimmu.2018.01626 (PMC6056728; doi:10.3389/fimmu.2018.01626)
Supplement: Supplementary file 4 [file Table_3.docx]

**Supplementary Table S3.** Shared DEGs in samples infected with wild-type and mutant reassortants.

| SoleaDB (v4.0/v4.1) unigene | Abbreviated  gene name | Log_2_ Fold change | | Coding protein | Related function |
| --- | --- | --- | --- | --- | --- |
|  |  | wSs160.03 | rSs160.03_247+270_ |  |  |
| Head-kidney | | | | | |
| 2256 | -- | -8.05 | -7.53 | Polyprotein n=1 Tax=Ljungan virus | -- |
| 226987 | ANXA3 | 3.10 | 3.00 | Annexin A3 | Regulation of cellular growth, signal transduction pathways |
| 226988 | ANXA3 | 3.29 | 2.99 | Annexin A3 | Regulation of cellular growth, signal transduction pathways |
| 2369 | COL1A2 | -5.13 | -5.22 | Collagen type I alpha 2 | Inflammatory response |
| 28986 | -- | 1.21 | 1.41 | Unassigned protein | -- |
| 324510 | LITAF | 1.27 | 2.13 | Lipopolysaccharide-induced tumor necrosis factor-alpha factor-like | Lysosome and NF-kappa B signaling |
| 429441 | ACTA1 | -6.17 | -4.19 | Cardiac muscle alpha actin 1 | Cell motility |
| 562801 | -- | -12.29 | -12.38 | Polyprotein n=1 Tax=Ljungan virus | -- |
| 630222 | NUP133 | 3.34 | 2.12 | Nucleoporin 133 | Interferon signaling and transport of the SLBP independent mature mRNA |
| 66200 | PTGES | -1.65 | -1.78 | Prostaglandin E synthase | Metabolism and eicosanoid synthesis |
| 70084_split_0 | ACTB | 3.14 | 2.07 | *Solea senegalensis* ACTB mRNA for beta actin isoform 1 | Cell motility, structure, and integrity |
| Eye/Brain | | | | | |
| 100120 | KLHL20 | -1.94 | -2.40 | Kelch-like protein 20-like | Immune system and E-cadherin signaling in the nascent adherents junction |
| 2369 | COL1A2 | -3.21 | -3.00 | Collagen type I alpha 2 | Inflammatory response |
| 27721 | ANGPTL7 | -2.33 | -2.86 | Angiopoietin-related protein 7 precursor | Response to oxidative stress |
| 35233 | PTCHD1 | -3.12 | -3.06 | Patched domain-containing protein 1 | Hedgehog receptor activity, involved in social behavior |
| 419148 | -- | -1.84 | -2.35 | Unknown | -- |
| 429349 |  | 3.18 | 2.49 | Peptidase_C2, Calpain family cysteine protease | Remodeling of cytoskeletal/membrane attachments, different signal transduction pathways, and apoptosis |

**Supplementary Table S3 (*continued*)**

| SoleaDB (v4.0/v4.1) unigene | Abbreviated  gene name | Log_2_ Fold change | | Coding protein | Related function |
| --- | --- | --- | --- | --- | --- |
|  |  | wSs160.03 | rSs160.03_247+270_ |  |  |
| Eye/Brain | | | | | |
| 43416 | SEMA3A | -1.40 | -1.20 | Semaphorin 3fa | Activation of cAMP-dependent PKA and GPCR pathway |
| 51411 | KIAA1199 | 2.10 | 2.72 | Cell migration inducing hyaluronan binding protein | Metabolism and glycosaminoglycan metabolism |
| 5200 | OBSCN | -1.23 | -1.14 | Obscurin-like | Signaling by GPCR and p75 NTR receptor-mediated signaling |
| 57524 | MCAM | -2.06 | -2.13 | Similar to melanoma cell adhesion molecule | Cell adhesion, and cohesion of the endothelial monolayer at intercellular junctions in vascular tissue |
| 6385 | -- | -1.41 | -1.54 | Similar to collagen XXI | Extracellular structural proteins involved in formation of connective tissue structure |
| 691154 | NDKA | -1.33 | -1.69 | Nucleoside diphosphate kinase A | Superpathway of pyrimidine deoxyribonucleotides *de novo* biosynthesis and metabolism |
