# Supplementary material for: Transcriptomic Profiles of Senegalese Sole Infected With Nervous Necrosis Virus Reassortants Presenting Different Degree of Virulence
Source: Front Immunol. 2018 Jul 17;9:1626. doi: 10.3389/fimmu.2018.01626 (PMC6056728; doi:10.3389/fimmu.2018.01626)
Supplement: Supplementary file 5 [file Table_4.docx]

**Supplementary Table S4**

Unigenes related to the most frequently detected ontologies within biological processes subclass in animals inoculated with both viral reassortants.

| Biological Process: Proteolysis GO:0006508 | | | | | | | |
| --- | --- | --- | --- | --- | --- | --- | --- |
| Sample | SoleaDB (v4.0/v4.1) unigene | FC | FDR | Solea-DB annotation | blast | ID (%)^a^ | Query Cover (%)^b^ |
| wSs160.03 | | | | | | | |
| Head-kidney | 26809 | 5.70 | 0.000661601 | Cathepsin S | *Lates calcarifer* cathepsin S-like | 83 | 44 |
|  | 325978 | 2.05 | 6.76146E-08 | Galectin-3-binding protein A precursor | *Paralichthys olivaceus* galectin 3 binding protein (lgals3bp) | 75 | 90 |
|  | 60137 | 8.35 | 3.58005E-05 | Zinc metalloproteinase nas-15-like isoform X4 | *P. olivaceus* zinc metalloproteinase nas-14-like | 77 | 69 |
|  | 60301 | 3.70 | 0.006071862 | Secreted trypsin-like serine protease | *P. olivaceus* chymotrypsin B-like | 80 | 85 |
|  | 6078 | 1.56 | 0.000884035 | Secernin-3 | *L. calcarifer* secernin 3 (scrn3), transcript variant X1 | 87 | 66 |
|  | 671015 | 9.04 | 0.000357807 | Cathepsin Z | *P. olivaceus* cathepsin Z-like | 86 | 86 |
|  | 69082 | 3.74 | 0.014589921 | Zinc metalloproteinase nas-15-like isoform X4 | *P. olivaceus* low choriolytic enzyme-like | 77 | 82 |
|  | 79207 | -1.67 | 0.024232104 | Coagulin factor II | *L. calcarifer* prothrombin-like | 81 | 94 |
|  | 7922 | 2.16 | 3.58101E-10 | Cathepsin L1 | *P. olivaceus* cathepsin L1-like | 83 | 66 |
| Eye/Brain | 14620_split_1 | 2.19 | 0.010568523 | Secreted trypsin-like serine protease | *L. calcarifer* suppressor of tumorigenicity 14 protein homolog | 84 | 93 |
|  | 28662 | 3.92 | 3.58849E-05 | Hatching enzyme | *Larimichthys crocea* uncharacterized LOC104932695 | 68 | 84 |
|  | 429349 | 3.18 | 0.001585923 | Peptidase_C2, Calpain family cysteine protease | *P. olivaceus* calpain-1 catalytic subunit-like | 73 | 84 |
|  | 430741 | 1.62 | 0.036685573 | Secreted trypsin-like serine protease | *L. calcarifer* transmembrane protease serine 4-like | 80 | 65 |
|  | 45874 | 3.03 | 0.003568653 | Transmembrane protease serine 9-like | *L. calcarifer* transmembrane protease serine 9-like | 75 | 75 |
|  | 45875 | 2.70 | 0.00323584 | Transmembrane protease serine 9-like | *L. calcarifer* transmembrane protease serine 9-like | 75 | 53 |
|  | 509203 | 2.58 | 0.004946421 | Calpain-1 catalytic subunit | *L. calcarifer* uncharacterized LOC108882260 | 85 | 77 |
|  | 6455 | 4.30 | 0.000272273 | Collagenase 3 | *P. olivaceus* macrophage metalloelastase-like | 81 | 73 |
|  | 95252 | 2.11 | 0.009006456 | Secreted trypsin-like serine protease | *L. calcarifer* suppressor of tumorigenicity 14 protein homolog | 85 | 52 |
| rSs160.03_247+270_ | | | | | | | |
| Head-kidney | 413926 | 2.46 | 0.043352018 | Elastase | *P. olivaceus* chymotrypsin-like elastase family member 2A | 81 | 58 |
|  | 429179 | 2.74 | 0.040001244 | Pancreatic elastase | *L. crocea* elastase-1-like | 83 | 85 |
|  | 429457 | 3.09 | 0.030436718 | High choriolytic enzyme 1 | *L. calcarifer* high choriolytic enzyme 1-like | 86 | 83 |
|  | 45873 | 2.73 | 0.00223158 | Unassigned protein | *L. calcarifer* transmembrane protease serine 9-like (LOC108878158), transcript variant X2 | 76 | 45 |
|  | 45874 | 2.50 | 0.020840849 | Transmembrane protease serine 9-like | *L. calcarifer* transmembrane protease serine 9-like | 75 | 75 |
|  | 45875 | 2.66 | 0.002819073 | Transmembrane protease serine 9-like | *L. calcarifer* transmembrane protease serine 9-like | 75 | 53 |
|  | 508186 | 2.65 | 0.041341384 | Trypsin | *Solea senegalensis* Tryp2 mRNA for trypsinogen 2 | 97 | 63 |
|  | 509093 | 2.87 | 0.005497145 | Secreted trypsin-like serine protease | *L. calcarifer* transmembrane protease serine 9-like (LOC108878158), transcript variant X2 | 77 | 81 |
|  | 509203 | 2.55 | 0.001073416 | Calpain-1 catalytic subunit | *L. calcarifer* uncharacterized LOC108882260 | 85 | 77 |
|  | 59663 | 3.00 | 0.034358666 | Chymotrypsin-like elastase family member 2A | *L. calcarifer* chymotrypsin-like elastase family member 2A | 80 | 58 |
|  | 627981 | 9.11 | 1.59624E-09 | Deleted in malignant brain tumors 1-like | *P. olivaceus* uromodulin-like | 85 | 87 |
|  | 93534 | 2.86 | 0.006372575 | Secreted trypsin-like serine protease | *L. calcarifer* transmembrane protease serine 9-like (LOC108878158), transcript variant X2 | 77 | 84 |
| Eye/Brain | 429349 | 2.49 | 0.017422916 | Peptidase_C2, Calpain family cysteine protease | *P. olivaceus* calpain-1 catalytic subunit-like | 73 | 84 |
|  | 4659 | -2.40 | 0.021638082 | Hedgehog protein | *Trachinotus ovatus* indian hedgehog B-like protein precursor | 87 | 81 |
|  | 73876 | -1.65 | 0.033052223 | Cathepsin H | *L. calcarifer* pro-cathepsin H-like (LOC108875435) | 82 | 70 |

| Biological Process: Vasculogenesis GO:0001570 | | | | | | | |
| --- | --- | --- | --- | --- | --- | --- | --- |
| Sample | SoleaDB (v4.0/v4.1) unigene | FC | FDR | protein | blast | ID (%) | Query Cover (%) |
| rSs160.03_247+270_ | | | | | | | |
| Eye/Brain | 15838 | -1.53 | 0.011319037 | Receptor-type tyrosine-protein phosphatase beta isoform X1 | *L. calcarifer* receptor-type tyrosine-protein phosphatase beta-like | 79 | 93 |
|  | 17400 | -2.00 | 0.015730606 | Protein HEG precursor | *L. calcarifer* heart development protein with EGF like domains 1 (heg1) | 82 | 83 |
|  | 19082 | -1.88 | 0.024602985 | EGF-like domain-containing protein 7 | *Monopterus albus* EGF like domain multiple 7 (egfl7), transcript variant X2 | 72 | 55 |
|  | 25583 | -1.87 | 0.032466978 | Protein tyrosine phosphatase | *L. calcarifer* receptor-type tyrosine-protein phosphatase eta-like (LOC108875356), transcript variant X5 | 77 | 89 |
|  | 462866 | -2.69 | 0.001504631 | Forkhead box protein C1 | *Cynoglossus semilaevis* forkhead box F2 (foxf2) | 89 | 88 |
|  | 5414 | -2.14 | 0.010722208 | Epidermal growth factor-like protein 7 precursor | *L. calcarifer* epidermal growth factor-like protein 7 (LOC108880572) | 79 | 90 |
|  | 7965 | -1.88 | 0.040353672 | Cerebral cavernous malformation 2-like | *L. calcarifer* CCM2 like scaffolding protein (ccm2l) | 89 | 81 |
|  | 8063 | -2.33 | 0.001736722 | Transcription factor Sox-7 | *L. calcarifer* transcription factor Sox-7-like (LOC108887411) | 85 | 73 |

^a^Percentage of nucleotide identity between query-subject alignment.

^b^Percentage of query covered by alignment to the database sequence.
