# Supplementary material for: Transcriptomic Profiles of Senegalese Sole Infected With Nervous Necrosis Virus Reassortants Presenting Different Degree of Virulence
Source: Front Immunol. 2018 Jul 17;9:1626. doi: 10.3389/fimmu.2018.01626 (PMC6056728; doi:10.3389/fimmu.2018.01626)
Supplement: Supplementary file 6 [file Table_5.docx]

**Supplementary Table S5**

Shared DEGs in different organs of Senegalese sole infected with each NNV.

| SoleaDB (v4.0/v4.1) unigene | Abbreviated  gene name | Log_2_ Fold change | | Coding protein | Related function |
| --- | --- | --- | --- | --- | --- |
|  |  | Head-kidney | Eye/brain |  |  |
| wSs160.03 | | | | | |
| 10919_split_0 | MACPF | 1.36 | 1.89 | Membrane attack complex component/perforin domain | Innate and adaptive immune responses. Complement-mediated cell lysis |
| 19559 | SMCHD1 | 3.61 | 2.35 | Structural maintenance of chromosomes flexible hinge domain-containing protein 1 | Chromosome organization |
| 19560 | SMCHD1 | 3.63 | 2.59 | Structural maintenance of chromosomes flexible hinge domain-containing protein 1 | Chromosome organization |
| 19849 | -- | 1.52 | 2.11 | Unknown | -- |
| 2061 | u-PAR | 2.89 | 2.87 | Urokinase plasminogen activator surface receptor-like protein | Regulates complement-mediated cell lysis. Lymphocyte signal transduction |
| 21834_split_0 | HERC5 | 3.80 | 2.53 | Probable E3 ubiquitin-protein ligase HERC5 | Mediates ISGylation of protein targets |
| 226987 | ANXA3 | 3.10 | 3.49 | Annexin A3 | Regulation of cellular growth. Signal transduction pathways |
| 226988 | ANXA3 | 3.29 | 2.96 | Annexin A3 | Regulation of cellular growth. Signal transduction pathways |
| 2369 | COL1A2 | -5.13 | -3.21 | Collagen type I alpha 2 | Inflammatory response |
| 2381 | ISG12 | 2.40 | 1.71 | Interferon-Stimulated Gene 12b Protein | Regulation of apoptosis |
| 2667 | STAT1 | 2.66 | 1.75 | Signal transducer and activator of transcription 1 | Mediates cellular responses to interferons (IFNs) |
| 280869 | -- | 3.03 | 3.18 | Unknown | -- |
| 281157 | Mx | 4.36 | 2.97 | Interferon-induced GTP-binding protein Mx | Antiviral response |
| 281655 | HERC5 | 3.72 | 2.69 | Probable E3 ubiquitin-protein ligase HERC5 | Mediates ISGylation of protein targets |
| 282251 | CCL19L1 | 2.82 | 2.95 | C-C motif chemokine ligand 19 like 1 | Involved in inflammatory and immunological responses. Lymphocyte recirculation and homing |
| 28297_split_2 | PARP14 | 1.55 | 1.53 | Poly [ADP-ribose] polymerase 14-like | Anti-apoptotic protein that may regulate aerobic glycolysis and promote survival of cancer cells |
| 30285 | STAT1 | 1.18 | 1.82 | Signal transducer and activator of transcription 1 | Mediates cellular responses to interferons (IFNs) |

**Supplementary Table S5** **(*continued*)**

| SoleaDB (v4.0/v4.1) unigene | Abbreviated  gene name | Log_2_ Fold change | | Coding protein | Related function |
| --- | --- | --- | --- | --- | --- |
|  |  | Head-kidney | Eye/brain |  |  |
| 30296 | STAT1 | 2.72 | 1.69 | Signal transducer and activator of transcription 1 | Mediates cellular responses to interferons (IFNs) |
| 324687 | u-PAR | 2.89 | 2.85 | Urokinase plasminogen activator surface receptor-like | Regulates complement-mediated cell lysis. Lymphocyte signal transduction |
| 326484 | EPSTI1 | 3.16 | 2.26 | Epithelial-stromal interaction protein 1-like | Cell proliferation |
| 326961 | DHX58 | 3.32 | 1.97 | DExH- box RNA helicase 58 | Involved in viral double-stranded (ds) RNA recognition and the regulation of immune response |
| 327171 | RTP3 | 2.64 | 2.64 | Receptor-transporting protein 3 | GPCR downstream signaling, olfactory signaling pathway, signal transduction, signaling by GPCR, virus responsive gene (VGR) in fish, immune response |
| 327265 | HERC4 | 4.14 | 2.57 | HECT And RLD Domain Containing E3 Ubiquitin Protein Ligase 4 | Immune system and Class I MHC mediated antigen processing and presentation |
| 328069 | HERC4 | 4.13 | 3.96 | HECT And RLD Domain Containing E3 Ubiquitin Protein Ligase 4 | Immune system and Class I MHC mediated antigen processing and presentation |
| 37410 | DHX58 | 3.32 | 2.27 | DExH- box RNA helicase 58 | Involved in viral double-stranded (ds) RNA recognition and the regulation of immune response |
| 3757 | RNF213 | 2.92 | 2.04 | RING finger protein 213-like | Immune system and Class I MHC mediated antigen processing and presentation |
| 4042 | HATPase_C | 3.01 | 3.39 | Histidine kinase-like ATPase, C-terminal domain | -- |
| 429349 | -- | 1.09 | 3.18 | Peptidase_C2, Calpain family cysteine protease | Remodeling of cytoskeletal/membrane attachments, different signal transduction pathways, and apoptosis |
| 44683 | IFIT1 | 3.53 | 2.68 | Interferon induced protein with tetratricopeptide repeats 1 | Inhibits viral replication and translational initiation |
| 44844 | -- | 2.51 | 1.61 | Unknown | -- |
| 462139 | -- | 2.07 | 2.53 | Uncharacterized protein LOC100332501 isoform X1 | -- |
| 46368 | HERC4 | 4.20 | 3.60 | HECT And RLD Domain Containing E3 Ubiquitin Protein Ligase 4 | Immune system and Class I MHC mediated antigen processing and presentation |

**Supplementary Table S5** **(*continued*)**

| SoleaDB (v4.0/v4.1) unigene | Abbreviated  gene name | Log_2_ Fold change | | Coding protein | Related function |
| --- | --- | --- | --- | --- | --- |
|  |  | Head-kidney | Eye/brain |  |  |
| 509076 | ISG12 | 2.39 | 2.36 | Interferon-Stimulated Gene 12b Protein | Regulation of apoptosis |
| 511960 | ZNFX1 | 4.32 | 1.90 | NFX1-type zinc finger-containing protein 1 | poly(A) RNA binding |
| 562832 | RTP3 | 5.17 | 4.01 | Receptor-transporting protein 3 | GPCR downstream signaling, olfactory signaling pathway, signal transduction, signaling by GPCR, virus responsive gene (VGR) in fish, immune response |
| 562986 | ISG15 | 4.62 | 2.72 | Interferon-stimulated gene 15 | Key role in the innate immune response to viral infection either via its conjugation to a target protein (ISGylation) or via its action as a free or unconjugated protein |
| 571269 | SACS | 3.58 | 2.38 | Sacsin-like | Chaperone binding and proteasome binding |
| 630222 | NUP133 | 3.34 | 3.39 | Nucleoporin 133 | Interferon signaling and transport of the SLBP independent mature mRNA |
| 64164 | MHC class II | 3.19 | 2.47 | Major histocompatibility complex class II integral membrane alpha chain precursor | Plays a central role in the immune system by presenting peptides derived from extracellular proteins |
| 6576_split_0 | TRIM21 | 3.10 | 2.44 | E3 ubiquitin-protein ligase TRIM21-like | Interferon signaling and immune system |
| 69090 | IFI44 | 2.64 | 2.45 | Interferon-induced protein 44-like protein | Response to virus, this protein aggregates to form microtubular structures |
| 70084_split_0 | ACTB | 3.14 | 3.96 | *Solea senegalensis* ACTB mRNA for beta actin isoform 1 | Cell motility, structure, and integrity |
| rSs160.03_247+270_ | | | | | |
| 231186 | RDH13 | -1.25 | -1.56 | Retinol dehydrogenase 13 | Signaling by GPCR and by retinoic acid |
| 2369 | COL1A2 | -5.22 | -3.00 | Collagen type I alpha 2 | Inflammatory response |
| 534198 | RPS12 | -1.63 | -1.90 | 40S ribosomal protein S12 | Viral mRNA translation. Activation of the mRNA upon binding of the cap-binding complex and eIFs, and subsequent binding to 43S and metabolism |
