# Supplementary material for: Transcriptomic Profiles of Senegalese Sole Infected With Nervous Necrosis Virus Reassortants Presenting Different Degree of Virulence
Source: Front Immunol. 2018 Jul 17;9:1626. doi: 10.3389/fimmu.2018.01626 (PMC6056728; doi:10.3389/fimmu.2018.01626)
Supplement: Supplementary file 7 [file Table_6.docx]

**Supplementary Table S6**

DEGs related to the immune response in Senegalese sole specimens inoculated with the wild-type VNNV-reassortant.

| Functional annotation | Gene name | Gene abbreviation (number of unigenes) | FC minimum-maximum | |
| --- | --- | --- | --- | --- |
| **Innate antiviral response** |  |  | Head-kidney | Eye/Brain |
| *Positive regulation of RIG-I signaling pathways* | Poly [ADP-ribose] polymerase 12 | PARP12 (2) | 1.7-1.84 |  |
| *Positive and negative regulation of RIG-I and MDA-5 signaling pathways* | DExH-box helicase 58 | DHX58 (2) | 3.32 | 1.97-2.27 |
| *Regulation of type I interferon (IFN)-dependent immune responses* | Interferon regulatory factor 3 | IRF3 (2) | 3.05-3.33 |  |
|  | Interferon regulatory factor 7 | IRF7 | 2.91 |  |
| *JAK-STAT cascade* | Signal transducer and activator of transcription 1-alpha/beta | STAT1 (4) | 2.62-2.72 | 1.69-2.67 |
|  | Interleukin 10 Receptor Subunit Beta | IL10RB | 1.62 |  |
| *Negative regulation of JAK-STAT cascade* | Suppressor of cytokine signaling 1 | SOCS1 | 2.53 |  |
| *Interferon signaling* | Interferon-stimulated gene 15 | ISG15 | 4.62 | 2.72 |
|  | Interferon-stimulated gene 12 | ISG12 (4) | 2.07-2.4 | 1.71-2.36 |
|  | Interferon-inducible protein Gig1 | Gig1(3) | 1.53-4.05 |  |
|  | Interferon-induced protein with tetratricopeptide repeats 1 | IFIT1 | 3.53 | 2.68 |
|  | IFN-induced protein 44 | IFI44 | 2.64 | 2.45 |
|  | Interferon-induced very large GTPase 1-like | VLIG1 | 2.67 |  |
|  | tripartite motif containing 21 | TRIM21 | 3.1 | 2.44 |
|  | Interferon- induced GTP-binding protein Mx | MxA | 4.36 | 2.97 |
|  | Double-stranded RNA-activated protein kinase R | PKR | 1.51 |  |
| *Protein ubiquitination* | E3 ubiquitin-protein ligase | HERC4 (3) | 4.13-4.2 | 2.57-3.96 |
|  | E3 ubiquitin-protein ligase | HERC5 (3) | 3.72-3.99 | 2.53-2.69 |
|  | Melanoma Antigen Family L2 | MAGEL2 | 11.08 |  |
| *Ubiquitin-dependent protein catabolic process* | Ubiquitin carboxyl-terminal hydrolase | USP18 (3) | 1.81-1.96 |  |
| *Antigen processing and presentation* | Gamma-Interferon-Inducible Lysosomal Thiol Reductase | GILT | 10.36 |  |
|  | ubiquitin-like modifier-activating enzyme 1 | UBE1 (2) | 1.99-2.1 |  |
|  | MHC Class II Antigen DRA | MHC class II alpha antigen | 3.19 | 2.47 |
|  | RING-Type E3 Ubiquitin Transferase | TRIM39 (4) | 3.89-4.29 | 3.32 |
|  | RING finger protein 213 | RNF213 (6) | 2.02-2.98 | 2.04 |
| *Virus responsive genes (VRG)* | CD20 domain containing protein | CD20 |  | 3.03 |
|  | Lily-type lectin-2 | Llec-2 (2) |  | 3.25-3.55 |
|  | Mannose-specific lectin | LMAN1(3) |  | 3.4-3.52 |
|  | Skin mucus lectin | Skin mucus lectin |  | 3.81 |
|  | Fish-egg lectin | Fish-egg lectin (2) |  | 4.37-4.47 |
|  | Galactoside-binding soluble lectin 9 | LGALS9 | 1.64 |  |
|  | Galectin 3 binding protein precursor | LGALS3BP (2) | 2.05-2.19 |  |
|  | Galectin 3 | LGALS3 (2) |  | 2.87-3.08 |
|  | Trypan-PARP multi-domain protein | Trypan-PARP |  | 2.03 |
|  | Receptor-transporting protein 3 | RTP3 (4) | 2.64-5.29 | 2.64-4.01 |
|  | Sacsin | SACS (2) | 3.43-3.58 | 2.38 |
|  | VHSV-induced protein | VHSV-IP | 2.52 |  |
|  | Herpes gp2 multi-domain protein | GP2 (2) | 15.35 | 3.42 |
|  | Claudin-like protein | ZF-A89 | 10.21 |  |
|  | Zinc-finger double-stranded RNA-binding | ZF-C3H7A | 1.69 |  |
|  | Sialic acid synthase | NANS(2) |  | 2.99-3.15 |
|  | Apolipoprotein D | APOD | 9.73 |  |
| *JNK signaling pathway* | Mitogen-Activated Protein Kinase Binding Protein 1 | MAPKBP1 | 2.35 |  |
| *Activation of MAP kinase cascade* | Proto-oncogene serine/threonine-protein kinase | MOS | 4.03 |  |
| *Regulation of inflammatory response* | Chemokine CCL-C5a precursor | CCL-C5a (3) | 2.12-2.31 |  |
|  | C-C motif chemokine ligand 19 | CCL19 | 2.82 | 2.95 |
|  | C-X-C motif chemokine 14 | CXCL14 | (-1.81) |  |
|  | CC chemokine CK3 | JFCCL4 (2) | 2.7-2.97 |  |
|  | C-X-C motif chemokine 25 | CXCL25 | (-2.91) |  |
|  | Cholecystokinin | CCK | 2.12 |  |
|  | C-C motif chemokine 28 | CCL28 |  | 3.57 |
|  | Interleukin 17 receptor C | IL17RC |  | 2.38 |
|  | Interleukin-27 subunit beta precursor | EBI3 | 2.3 |  |
|  | u-PAR/Ly-6 domain | u-PAR/Ly-6 (3) |  | 4.33-4.47 |
|  | 4F2 cell-surface antigen heavy chain-like | SLC3A2 | 9.9 |  |
| *Immune effectors* | Complement factor H precursor | CFHR3 (4) | (-1.98)-(-3.01) |  |
|  | Macrophage expressed 1-Membrane attack complex component/perforin domain | MACPF |  | 1.89 |
|  | NFX1-type zinc finger-containing protein 1 | ZNFX1 | 4.32 | 1.9 |
|  | Cathepsins | Cathepsins L1, S, Z (3) | 2.16-9.04 |  |
|  | Cytochrome b-245 alpha polypeptide | CYBA |  | 1.81 |
|  | Urokinase plasminogen activator surface receptor | u-PAR (2) | 2.89 | 2.85-2.87 |
| **Adaptative immune response** |  |  | Head-kidney | Eye/Brain |
| *Immunosuppression and regulation of anti-tumor activity* | OX-2 membrane glycoprotein | CD200 | 1.78 |  |
| *Regulation of T-cell proliferation and lymphotoxin signaling* | T-cell immunoglobulin and mucin domain-containing protein 4 | TIMD4 | 1.84 |  |
|  | Eomesodermin | EOMES | 2.21 |  |
| *Regulation of apoptosis* | Baculoviral IAP Repeat Containing 5 | BIRC5 | 11.22 |  |
|  | Death Associated Protein Like 1 | DAPL1 | 2.48 |  |
|  | rho GTPase-activating protein 11A | ARHGAP11A | 7.75 |  |
|  | GTPase IMAP family member 8 | GIMAP8 (2) |  | 1.61-1.76 |
|  | v-ets erythroblastosis virus E26 oncogene homolog | ETS1 | 8.27 |  |
|  | Gelsolin | GSN (7) |  | 3.29-4.58 |
|  | BCL2 Interacting Protein 2 | BNIP-2 | 2.05 |  |
|  | Leukocyte elastase inhibitor | SERPINB1 (3) |  | 2.44-4.69 |
|  | Plakophilin-1 | PKP1 (2) |  | 2.19-2.2 |
