# Supplementary material for: Transcriptomic Profiles of Senegalese Sole Infected With Nervous Necrosis Virus Reassortants Presenting Different Degree of Virulence
Source: Front Immunol. 2018 Jul 17;9:1626. doi: 10.3389/fimmu.2018.01626 (PMC6056728; doi:10.3389/fimmu.2018.01626)
Supplement: Supplementary file 8 [file Table_7.docx]

**Supplementary Table S7**

DEGs related to the immune response of Senegalese sole specimens infected with the mutant VNNV-reassortant isolate

| *S. senegalensis*  transcriptome  unigene | Gene | Short name | Log-FC  Head Kidney | Log-FC  Eye/Brain | | Associated function |
| --- | --- | --- | --- | --- | --- | --- |
| 23693 | TRAF3-interacting JNK-activating modulator-like | TRAF3IP3 | -- | | -1.6 | The gene encodes a protein that mediates cell growth by modulating the JNK signal transduction pathway |
| 65337 | Tyrosine protein kinase | BTK | -- | | -1.8 | Plays an important role in the function of immune cells of innate as well as adaptive immunity, as a component of the Toll-like receptors (TLR) pathway |
| 510055 | E3 ubiquitin-protein ligase TRIM39-like | TRIM39 | 2.3 | | -- | Signaling pathways |
| 284611 | Tripartite motif-containing protein 47 | FTR82 | -- | | -1.5 | Signaling pathways. Fish virus induced TRIM protein |
| 19572 | RING-finger-containing E3 ubiquitin ligase | FTR26 | 2.1 | | -- | Signaling pathways. Fish virus induced TRIM protein |
| 45763_split_1 | Interleukin 17 receptor E | IL17RE | -- | | -1.6 | Inflammatory response |
| 200319 | Regulator of G-protein signaling 5 | RGS5 | -- | | -2.7 | Signaling pathways |
| 28120 | Chemokine (C-X-C motif) ligand 12a (stromal cell-derived factor 1) precursor | CXCL12 | -- | | -1.8 | Inflammatory response |
| 229367 | CXC chemokine receptor 4 | CXCR4 | -- | | -2.1 | Inflammatory response |
| 43142 | CXC chemokine receptor 7b | CXCR7 | -- | | -1.6 | Inflammatory response |
| 57524 | Melanoma cell adhesion molecule (MCAM) | CD146 | -- | | -2.1 | Signaling pathways and cell proliferation |
| 52152 | Tetraspanin-7 | TSPAN7 | -- | | -1.4 | Regulate cell migration , fusion, and signaling events |
| 42274 | Tetraspanin-8 | TSPAN8 | 1.8 | | -- | Regulate cell migration , fusion, and signaling events |
| 40976 | T-cell-specific surface glycoprotein CD28 | CD28 | -- | | -2.1 | Innate immune system. Essential co-stimulatory receptor critical for activation, proliferation and survival processes in CD4+ T cells |
| 323999_split_1 | Scavenger receptor cysteine-rich type 1 protein M130-like | CD163 | 8.7 | | -- | May play an anti-inflammatory role. Macrophage activation in inflammatory conditions |
| 46995 | Carcinoembryonic antigen-related cell adhesion molecule 5 precursor | CD66e | 5.3 | | -- | Cell surface glycoprotein that plays a role in cell adhesion and in intracellular signaling |
| 22112 | Natural Killer Cell Cytotoxicity Receptor 3 Ligand 1 | NCR3LG1 | 7.9 | | -- | Selectively expressed on tumor cells. Natural killer (NK) cell activation and cytotoxicity |

**Supplementary Table S7 (*continued*)**

| *S. senegalensis*  transcriptome  unigene | Gene | Short name | Log-FC  Head Kidney | Log-FC  Eye/Brain | | Associated function |
| --- | --- | --- | --- | --- | --- | --- |
| 22753 | Monocyte to macrophage differentiation factor 2-like | MMD2 | 4.3 | | -- | Cell proliferation and inflammatory response |
| 690851 | Macrophage mannose receptor 1 | ISP2 | 10.6 | | -- | Complement activation |
| 282448 | Complement component C3 | C3 | 3.2 | | -- | Complement system and contributes to innate immunity |
| 57917 | Immunoglobulin superfamily member 11 precursor | IGSF11 | 6.2 | | -- | Cell adhesion molecule |
| 60160 | Preprospasmolysin | FIM-A.1 | 3.6 | | -- | Defense against microbial infections |
| 627981 | Deleted in malignant brain tumors protein 1-like | DMBT1  (CD5L) | 9.1 | | -- | Tumor suppressor gene. May play roles in mucosal defense system and cellular immune defense. |
| 280451 | Epidermis-type lipoxygenase 3 | ALOXE3 | -- | | -3.05 | Leukotriene metabolic process |
| 602054 | Polymeric immunoglobulin receptor | PLGR | -- | | -1.8 | Fc receptor which facilitates the secretion of the soluble polymeric isoforms of immunoglobulin A and immunoglobulin M |
| 58274 | Prostate Stem Cell Antigen-like | PSCA | 11.6 | | -- | May be involved in the regulation of cell proliferation |
| 98446 | T-cell differentiation protein | MAL | 2.2 | | -- | T-cell signal transduction |
| 4523 | B-cell CLL/lymphoma 6, member B-like | BCL6B | -- | | -1.7 | B-cell development |
| 58626 | GTPase IMAP family member 4-like | GIMAP4 | -- | | -1.6 | May play a role in regulating lymphocyte apoptosis |
| 284340 | Complement C1q tumor necrosis factor-related protein 9-like | C1QTNF9B | -- | | -2 | Probable adipokine. Activates AMPK, AKT, and p44/42 MAPK signaling pathways |
| 67484 | Hemoglobin alpha chain | HBA | -- | | -2.1 | Pro-apoptotic factor in programmed cell death |
| 686713 | Hemoglobin subunit beta-1 | HBB1 | -- | | -2.2 | Oxygen transport |
| 45875 | Secreted trypsin-like serine protease | PRSS27 | 2.7 | | -- | Immune response |
| 684964 | CLECT domain containing protein | CTL | 10.2 | | -- | C-type lectin. Recognition molecules within the immune system, their functions involving defense against pathogens, cell trafficking, immune regulation and the prevention of autoimmunity |
| 520631 | IgGFc-binding protein-like | FCGBP | 5.1 | | -- | May play an important role in immune protection and inflammation |
